# Distribution of Phenolic Compounds and Antioxidant Activity in Plant Parts and Populations of Seven Underutilized Wild *Achillea* Species

**DOI:** 10.3390/plants11030447

**Published:** 2022-02-06

**Authors:** Lina Raudone, Jolita Radušiene, Fatih Seyis, Fatih Yayla, Gabrielė Vilkickyte, Mindaugas Marksa, Liudas Ivanauskas, Cüneyt Cırak

**Affiliations:** 1 Department of Pharmacognosy, Lithuanian University of Health Sciences, Sukileliu Av. 13, 50162 Kaunas, Lithuania; 2 Laboratory of Biopharmaceutical Research, Institute of Pharmaceutical Technologies, Lithuanian University of Health Sciences, Sukileliu Av. 13, 50162 Kaunas, Lithuania; gabriele.vilkickyte@lsmu.lt; 3 Institute of Botany, Nature Research Center, Akademijos Str. 2, 08412 Vilnius, Lithuania; jolita.radusiene@gamtc.lt; 4 Department of Field Crops, Faculty of Agriculture and Natural Sciences, Recep Tayyip Erdoğan University, 53100 Rize, Turkey; fatih.seyis@erdogan.edu.tr; 5 Department of Biology, Faculty of Arts and Sciences, Gaziantep University, 27310 Gaziantep, Turkey; fyayla@gantep.edu.tr; 6 Department of Analytical and Toxicological Chemistry, Lithuanian University of Health Sciences, Sukileliu Av. 13, 50162 Kaunas, Lithuania; mindaugas.marksa@lsmuni.lt (M.M.); liudas.ivanauskas@lsmuni.lt (L.I.); 7 Bafra Vocational School, Ondokuz Mayis University, Atacum, 55200 Samsun, Turkey; kalinor27@gmail.com

**Keywords:** *Achillea* species, phenolic profile, antioxidant activity, interspecific diversity

## Abstract

Evaluation of phytochemical composition of underutilized *Achillea* species provides the primary selection of germplasms with the desired quality of raw material for their further applications. The aim of the study was to evaluate the comprehensive distribution patterns of phenolic compounds in seven wild *Achillea* spp. and their plant parts, and to assess their antioxidant activity. Plant material was collected from different sites in Turkey. A complex of hydroxycinnamic acids, flavonols and flavones was identified and quantified in methanolic extracts using HPLC-PDA method. Antioxidant activity was assessed by radical scavenging assay. The results showed that qualitative and qualitative profiles of caffeoylquinic acids and flavonoids were species-specific, explaining the characteristic patterns of their variation in the corresponding species and plant parts. The highest total amount of caffeoylquinic acids was detected in *A. setacea*. *A. arabica* exposed the highest accumulation of mono-caffeoylquinic acids and flavonoids with the greatest levels of quercetin and luteolin derivatives and the flavonol santin. Santin was detected in all plant parts of *A. cappadocica*, *A. setacea*, *A. santolinoides* subsp. *wilhelmsii*, and *A. arabica*. A notable antiradical capacity was confirmed in *A. arabica*, *A. setacea* and *A. cappadocica* plant extracts. The leaves of all studied species were found to have priority over inflorescences and stems in terms of radical scavenging activity. The new data complemented the information that may be relevant for the continuation of chemophenetic studies in the heterogeneous genus *Achillea*.

## 1. Introduction

The increasing trend in use of natural products or natural product derivatives is leading to a growing demand for standardized, homogeneous raw materials in industry, which means that more and more wild species have been domesticated and systematically cultivated. On the other hand, the International Standard for Sustainable Wild Collection of Medicinal and Aromatic Plants (ISSC-MAP) certifies the conservation and use of plant biodiversity, avoiding overharvesting leading to genetic erosion and habitat loss, but these provisions have been considered in the context of economically sustainable use of plant resources. The selection of germplasm from wild populations with commercially desirable traits is the primary approach that can provide opportunities for the selection and development of medicinal plants as crops, as well as to reduce harvesting pressure on wild populations. Cultivated plant material has a number of advantages over wild harvest, in particular reliable botanical identification, a narrow genetic range, and the quality control and standardization of materials can be adapted to the regulations and consumer preferences [1]. Therefore, the assessment of phytochemical composition of wild plant raw material sources is the reason of the primary selection of economically important medicinal plant germplasms with the desired quality of raw material for their further domestication and cultivation.

The genus *Achillea* L. of the Asteraceae family, depending on the species definition, includes about 110–140 species [2] and provides a high potential of underutilized plant resources. Turkey is the largest *Achillea* species distribution region with 69 identified species, 33 of which are endemic [3]. *Achillea* species are one of the oldest medicinal plants used in folk and traditional medicine in various countries as a natural remedy for the treatment of wounds, bleedings, gastrointestinal disorders, headache, inflammation, pains, and spasmodic diseases [4]. The use of *Achillea* spp. is highly important in Persian and Anatolian folk herbal medicines for the treatment of various disorders [5]. The ethnopharmacological information is further developed in the selection of species with the potential to be used in pharmaceuticals. Despite the great diversity of *Achillea* spp., only *A. millefolium* L., commonly known as “yarrow”, is worldwide recognized as a medicinal raw material. Plant material of flowering tops (*Millefolii herba*) is recorded in a number of monographs of the European National Pharmacopoeias and described in the report of the European Medicines Agency as an herbal medicine [6,7]. In this way, the growing industrial demand for raw materials is a major challenge to explore the wide diversity of underutilized *Achillea* spp. and to find alternative sources that have could be valuable in the pharmaceutical, cosmeceutical or food industries. There is strong scientific evidence that other species of yarrow might be rich sources of bioactive ingredients. Scientific experimental and clinical studies have confirmed the various therapeutic activities in different *Achillea* species such as antimicrobial, anti-inflammatory, cytotoxic, antinociceptive, anti-inflammatory, wound healing, estrogenic, and antispasmodic, hepatoprotective and choleretic. [8].

*Achillea arabica* Kotschy, together with *A. millefolium*, is the most common species of genus in Turkish flora. In most of the previous sources, *A. arabica* was referred by a taxonomic synonym for *A.* biebersteinii Afan. ex Hub.-Mor., which was described later, so the first name for the corresponding taxon, A. arabica, was adopted [9]. The therapeutic potency of *A. arabica* as a medicinal plant has been recognized and documented in various pharmacological studies as an alternative in the treatment of endometriosis [10], wound healing [10,11,12], and was considered as a promising anticancer agent for the treatment of colon cancer [13]. *A. biebersteinii* hydroglycolic extract has been displayed significant antiradical, tyrosinase inhibiting and sun protective properties as an active ingredient in cosmetics [14]. *A. wilhelmsii* K.Koch, a synonym of accepted name *A. santolinoides* subsp. *wilhelmsii* (K.Koch) Greuter [15], has been shown to have antihyperlipidemic, antihypertensive, and antimycobacterial properties [16], and has potential for the treatment of indomethacin-induced gastric ulcers [17]. The treatment with *A. wilhelmsii* hydroalcoholic extract significantly reduced triglycerides, total cholesterol, and low-density lipoproteins, and reduced diastolic and systolic blood pressure [18]. *A. wilhelmsii* leaf and stem extracts induced antiproliferative and apoptotic effects on prostate cancer cell lines [19]. Gevrenova et al. [20] detected that extracts of *A. santolinoides* and *A. allepica* DC. areal parts and roots significantly inhibited butyrylcholinesterase and tyrosinase, suggesting the potential applications of these plants in cosmetics, pharmaceuticals, and nutraceuticals products. *A. coarctata* Poir. extracts showed cytotoxic activity on human breast cancer cell lines, while essential oil had antimicrobial activity against several pathogenic microorganisms [20,21]. The lypophilic extract of *A. setacea* showed hemostatic activity in the case of toxic animal hepatitis [22]. A literature survey revealed that there is only one report on the endemic *A. cappadocica* Hausskn. & Bornm., which showed some antioxidant, anticholinesterase, antimicrobial activity of the extract [23]. Numerous studies on the composition and biological activities of essential oils have been performed on *A. biebersteinii*, *A. wilhelmsii, A. setacea*, and *A. coarctata* [21,24,25]. The biological and pharmacological activities of *Achillea* spp. were mainly attributed to the variable compounds of essential oils and polyphenolic compounds with the highest importance of azulenogenous sesquiterpene lactones, flavonoids and phenolic acids [26,27]. Moreover, the chemical profile and biological effects of different species and their plant parts vary greatly [28]. Therefore, it is of crucial importance to identify differences in extracts to obtain consistent and targeted biological test results. Recently, much attention has been paid to towards phenolic acids and flavonoids, which are considered to be high important biologically active compounds in *Achillea* spp. with strong antioxidant effects [29,30,31,32]. However, data on the qualitative and quantitative phenolic profiles of *Achillea* spp. are scarce. Moreover, the chemical profiles and biological effects of raw materials vary greatly between different species and their plant parts [28]. Therefore, it is essential to identify differences in the profiles of plant extracts to obtain consistent and validated results for further biological tests. The study aimed to provide a comparative assessment of the diversity of phenolic profiles and their antioxidant activity of seven underutilized *Achillea* spp. for reasonably further evaluation by selecting the desired germplasm. To the best of our knowledge, the comprehensive patterns for the distribution of phenolic compounds in wild populations of seven *Achillea* spp. and their plant parts have been determined for the first time. The obtained results will complement the knowledge on phenolic profiles and species diversity in the genus *Achillea*.

## 2. Results

### 2.1. Phenolic Profiles of Leaves, Inflorescences, and Stems across Achillea Species

A complex of hydroxycinnamic acids, flavonols and flavones was detected in the leaves, inflorescences, and stems of the seven wild *Achillea* species (Table 1, Table 2 and Table 3). Chemical profiles of *Achillea* spp. varied according to the amounts of individual compounds and their distribution among plant parts. Significant differences were found in the accumulation of all compounds among the seven species and their leaves, inflorescences, and stems, with the exception of neochlorogenic acid in the leaves and inflorescences, apigenin in the leaves and 3.5-dicaffeoylquinic acid in the stems. On average, caffeoylquinic acids comprised about 77% of total identified phenolic compounds in the profiles of wild *Achillea* species. The highest total amount of all identified caffeoylquinic acids was detected in *A. setacea* leaf, inflorescence, and stem samples, 25,842.38, 11,913.84 and 4696.72 µg/g, respectively. Differently from other species, *A. arabica* accumulated higher amounts of flavonoids than caffeoylquinic acids. The amount of total identified caffeoylquinic acids in the leaf and inflorescence samples of investigated species can be presented in the following decreasing order: *A. setacea > A. arabica* ~ *A. coarctata* > *A. aleppica* > *A. cappadocica* > *A. santolinoides* > *A. santolinoides* subsp. *wilhelmsii*. However, the amounts of caffeoylquinic acid in stem samples did not follow this trend. Moreover, caffeoylquinic acids, namely chlorogenic, 3,4-dicaffeoylquinic acid, 3,5-dicaffeoylquinic acid and 4,5-dicaffeoylquinic acid, were the predominant compounds in leaves, inflorescences, and stems, with significant quantitative changes between species. Significantly the highest (*p* < 0.05) content of chlorogenic acid was found in *A. arabica* leaves and *A. setacea* inflorescences, 3,5-dicaffeoylquinic acid predominated in *A. cappadocica* and *A. setacea* leaf and inflorescence samples, while 3,4-dicaffeoylquinic acid prevailed in all parts of *A. setacea*. Meanwhile, the significantly highest content of 4,5-dicaffeoylquinic acid was detected in *A. coarctata* leaf and inflorescence samples. In addition, more notable levels of neochlorogenic acid were detected in the leaves of *A. aleppica* and *A. arabica*. *Achillea* spp. accumulated minor contents of 1,5-O-dicaffeoylquinic and caffeic acids in all plant parts. Consequently, depending on the prevailing caffeoylquinic acid, the species can be divided into three groups. One group combined *A. aleppica*, *A. santolinoides* subsp. *wilhelmsii*, and *A. arabica* species, which were dominated by chlorogenic acid. Meanwhile, *A. cappadocica*, *A.setacea*, and *A. santolinoides* were prevailed by 3,5-dicaffeoylquinic acid, while *A. coarctata* was dominated by both 4,5-dicaffeoylquinic and 3,5-dicaffeoylquinic acids. Thus, the studied species differed in their quantitative profiles of mono- and dicaffeoylquinic acids. The mode of species-specific profiles of caffeoylquinic acids showed significant quantitative differences between plant parts, the amount of which predominated in the leaves. The quantitative variation of caffeoylquinic acids in the stems correspondent weakly to the patterns determined in the leaves and inflorescences, nevertheless, chlorogenic acid and 3,5-dicaffeoylgquinic acid remained the predominant compounds in all stems of *Achillea* spp. tested. 

The flavonol complex consisted of quercetin and its derivatives, namely quercitrin, rutin, isoquercitrin, and methylated flavonol santin. Notable amounts of quercitrin were detected in *A. arabica* leaf, inflorescence, and stem samples, accounting for 8904.9 ± 5126.48 µg/g, 8487.21 ± 3445.28 µg/g and 2442.38 ± 1407.88 µg/g, respectively, whereas other species did not accumulate this compound at all. Santin was detected in all plant parts of *A. cappadocica*, *A. setacea*, *A. santolinoides* subsp. *wilhelmsii*, and *A. arabica*. Leaf and inflorescences of *A. setacea* differed significantly with the highest amounts of santin, 784.02 ± 287.25 µg/g and 548.80 ± 190.9 µg/g, respectively. Meanwhile, santin was not detected in *A. aleppica*, *A. coarctata* and *A. santolionides*. Rutin was found in all plant parts of all tested species, except for *A. aleppica* raw materials, in which this compound was not detected. The amounts of isoquercitrin varied significantly within the species and their plant parts, with the highest levels in *A. arabica* inflorescences and leaves.

The identified flavones were luteolin, apigenin and their glucosides. Luteolin-7-glucoside was the only flavone compound found in all species tested and their plant parts. The highest levels of this compound were accumulated in *A. arabica*, which ranged from 198.55 in stems to 5646.13 µg/g in inflorescences. The highest amounts of luteolin derivatives were found in the inflorescences of *A. arabica*, *A. santolinoides*, and *A. setacea*. Overall, the pattern of caffeoylquinic acid and flavonoid profiles showed significant quantitative differences between species and plant parts that predominated in the leaves compared to the inflorescences and stems. All species except *A. arabica* were dominated by accumulation of caffeoylquinic acids, with the highest total content in *A. setacea*. *Achillea setacea*, *A. coarctata* and *A. cappadocica* were prevailed by dicaffeoylquinic acids, while *A. arabica* and *A. aleppica* by mono-caffeoylquinic acids. *Achillea arabica* was distinguished by an exceptional quantitative profile of flavonoids, with the highest content of quercetin and luteolin derivatives compared to other species. In addition, *A. arabica* raw materials distinguished the highest total amount of all detected compounds, averaging 23,511.9, 38,180.5, and 8124.4 µg/g, in leaf, inflorescence and stem samples, respectively. Meanwhile, *A. aleppica* plant raw materials accumulated the lowest amounts of all identified flavonoids. 

### 2.2. Antioxidant Activity of Leaves, Inflorescences, and Stems of Wild Achillea Species

**Figure 1:**
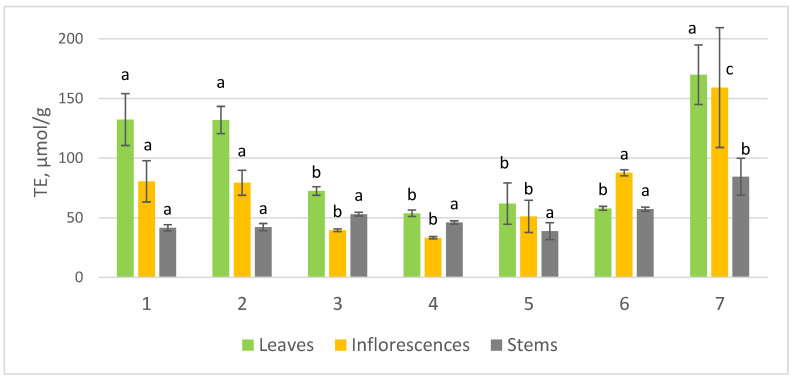
Variation of mean antioxidant activity (TE, µmol/g) in plant parts of seven wild *Achillea* species: *A. cappadocica* (1); *A. setacea* (2); *A. aleppica* (3); *A. coarctata* (4); *A. santolinoides* subsp. *wilhelmsii* (5); *A. santolinoides* (6); *A. arabica* (7). The columns marked with the different letters (a, b, c) were significantly differed at *p* ≤ 0.05 among species according to the Duncan’s Multiple Range test.

The results on antioxidant activity analysis revealed significant differences (*p* ≤ 0.05) among species and plant parts (Figure 1). The samples of *A. arabica* were elucidated with the highest TE values among all tested materials, 169.89 ± 24.93, 159.11 ± 50.25 and 84.39 ± 15.50 µmol/g, in leaves, inflorescences and stems, respectively. The leaves of all studied species were found to have priority over inflorescences and stems in terms of radical scavenging activity, with the exception for *A. santolinoides*, where no significant difference was found between the leaf and stem samples. The TE values of *A. cappadocica* and *A. setacea* leaf extracts were higher than 130 µmol/g and together with *A. arabica* showed the significantly highest (*p* < 0.05) antioxidant activity compared to the other species studied. Meanwhile, *A. arabica* was superior to other species in terms of the highest mean TE value in inflorescences. *A. santolinoides*, *A. cappadocica*, and *A. setacea* were superior in radical scavenging activity with the mean values of TE exeeding 79 µmol/g. The leaves and inflorescences of *A. aleppica*, *A. coarctata* and *A. santolinoides* subsp. *wilhelmsii* were found to have comparable radical scavenging activity. No significant differences in antioxidant activity were found among the stems, except for A. arabica, whose stems differed in the higherst antioxidant activity (84.39 ± 15.50 µmol/g, *p* < 0.05) compared to other species. The relationship between radical scavenging activity and population growing site in terms of its elevation was verified using Pearson correlational analysis. However, no significant correlations were found between the variables. Meanwhile, the radical scavenging activity of plant extracts showed a statistically significant correlation with the contents of some individual phenolics. There was a positive correlation between the contents of neochlorogenic acid (r = 0.545, *p* < 0.0001), chlorogenic acid (r = 0.473, *p* < 0.0001), 3,5-dacaffeoylquinic acid (r = 0.310, *p* < 0.0001), quercitrin (r = 0.663, *p* < 0.0001), quercetin (r = 0.527, *p* < 0.0001), and luteolin-7-glucoside (r = 0.371, *p* < 0.0001) and their antioxidant activities. 

Consequently, *Achillea* spp. exposed considerable antioxidant activity, which differed among species and plant parts in parallel to their individual phenolic compound contents. The high abundance of phenolics in *A. arabica*, *A. setacea* and *A. cappadocica* have induced a significant effect on the radical scavenging activity, with the greatest possible contribution of chlorogenic acid and quercetin derivatives. The leaves of all studied species were superior to inflorescences and stems in terms of radical scavenging activity.

### 2.3. Intra- and Interspecific Differences; Principal Component Analysis 

Principal component analysis (PCA) was performed to elucidate the intra- and interspecific differences in seven wild *Achillea* spp. according to the compound levels in their plant parts. Individual phenolic compounds were used as variables to create three-dimensional PCA square matrix models to visualize the available intra-and interspecific patterns of *Achillea* spp. phenolic profiles in their plant parts.

**Figure 2:**
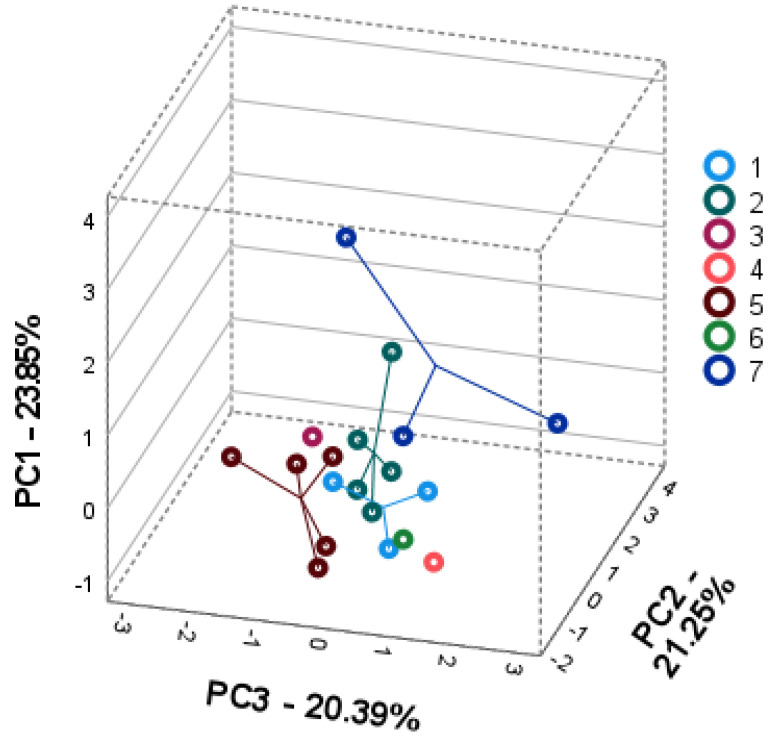
PCA1 score plots model representing the accumulation of phenolic compounds in leaves of seven *Achillea* species: *A. cappadocica* (1); *A. setacea* (2); *A. aleppica* (3); *A. coarctata* (4); *A. santolinoides* subsp. *wilhelmsii* (5); *A. santolinoides* (6); *A. arabica* (7).

The PCA1 score plot correlation matrix explained 65.52% of the total variance of the leaf data set model (Figure 2). PC1 accounted for 23.85% of the total data set and was positively correlated with mono-caffeoylquinic acids (neochlorogenic (0.969) and chlorogenic (0.819)) and quercitrin (0.868), and negatively with luteolin (−0.544). PC2 explained 21.25% of the total variance and was highly positively correlated with 3,4-dicaffeoylquinic acid (0.859), 3,5-dicaffeoylquinic acid (0.938), and santin (0.807). PC3 accounted for 20.39% of the total data set variance and was associated with rutin (0.861), isoquercitrin (0.709), luteolin-7-glucoside (−0.628) and luteolin-7-rutinoside (−0.511). The PCA1 score plot (Figure 2) showed the arrangement and grouping of populations of seven *Achillea* spp. tested. Populations of *A. arabica* (no 7) showed a distant position from other species, which can be explained by the high content of mono-caffeoylquinic acids and quercitrin in the leaves of this species. *A. santolinoides* subsp. *wilhelmsii* (no 5) populations clustered along negative PC2 and PC3 due to the high values of luteolin derivatives. Populations of *A. cappadocica* (no 1), *A. setacea* (no 2), and *A. santolinoides* (no 6) formed an overlapping group distinguished by a high quantitative similarity in the accumulation of 3,4- and 3,5-dicaffeoylquinic acids. The position of *A. coarctata* (no 4) can be explained by the contribution of rutin with a high positive PC3 loading.

**Figure 3:**
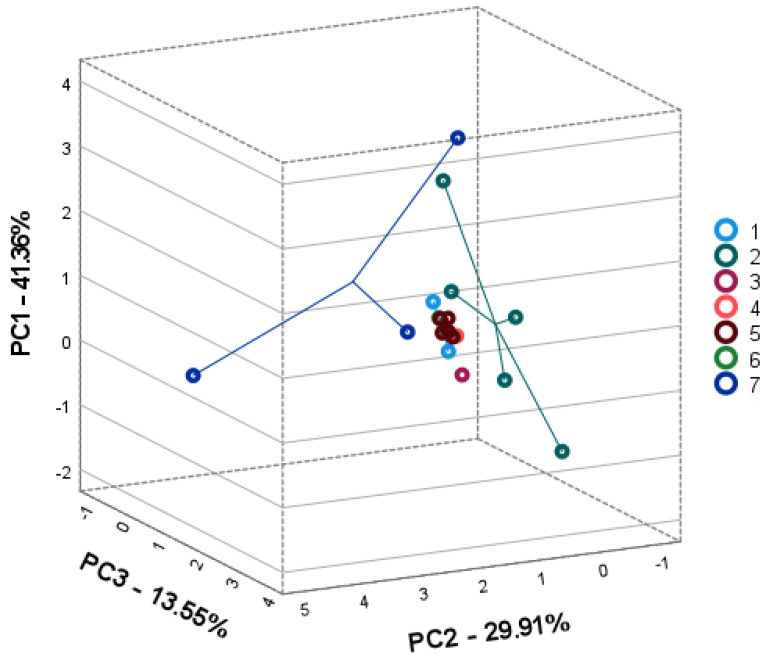
PCA2 score plots model representing the accumulation of phenolic compounds in inflorescences of seven *Achillea* species: *A. cappadocica* (1); *A. setacea* (2); *A. aleppica* (3); *A. coarctata* (4); *A. santolinoides* subsp. *wilhelmsii* (5); *A. santolinoides* (6); *A. arabica* (7).

The PCA2 score plot correlation matrix explained 84.81% of the total variance of the inflorescence data set model (Figure 3). PC1 accounted for 41.36% of the total variance and highly correlated with variables, namely rutin (0.613), isoquercitrin (0.530), luteolin (0.902), luteolin-7-glucoside (0.648), luteolin-7-rutinoside (0.831), luteolin-3,7-diglucoside (0.922), and santin (0.556). PC2 explained 29.91% of the total variance and of the data set showing high correlation with neochlorogenic acid (0.982), quercitrin (0.957), rutin (0.680), and quercetin (0.976). PC3 accounted for 13.55% of the total variance and correlated with the variables, namely chlorogenic acid (0.907), 3,4-dicaffeoylqionic acid (0.911), 3,5-dicaffeoylquinic acid (0.925), and isoquercitrin (0.614). The clustering of inflorescence differed from the leaf grouping pattern (Figure 3). The inflorescences of *A. cappadocica* (no 1), *A. coarctata* (no 4), *A. santolinoides* subsp. *wilhelmsii* (no 5), and *A. santolinoides* (no 6) were clustered in a close group along the negative PC2 and PC3 with high contributions of chlorogenic acid, 3,4-and 3,5-dicaffeoylquinic acids, and isoquercitrin. *A. aleppica* (no 3) positioned apart from the abovementioned cluster of species, at lower PC1 position with the contribution with the contribution of the lowest flavonoid contents. *A. setacea* (no 2) and *A. arabica*) (no 7) populations were highly scattered on the PCs score plots displaying a higher variation of bioactive compounds than other species.

**Figure 4:**
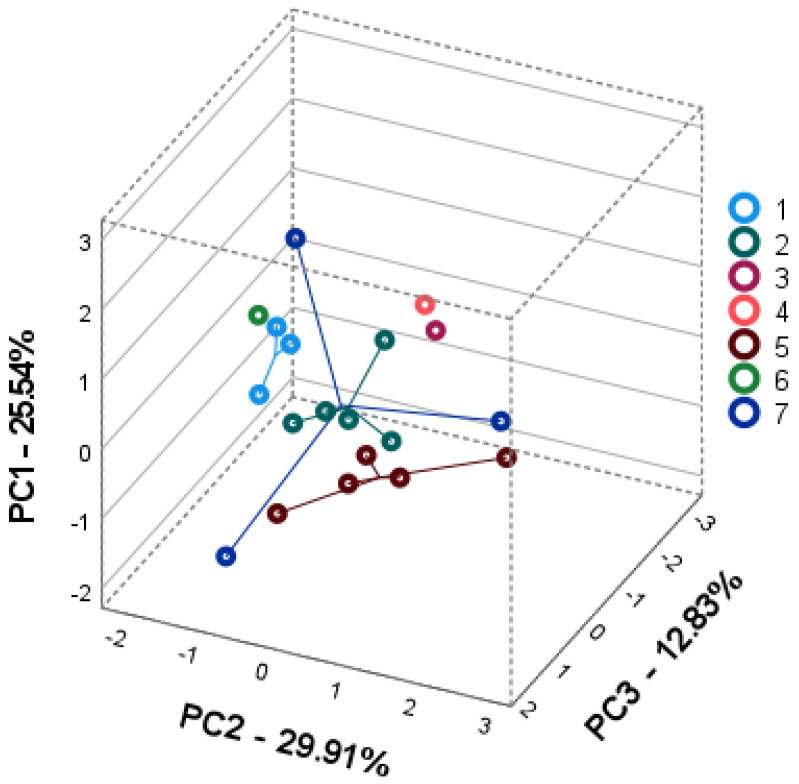
PCA2 score plots model representing the accumulation of phenolic compounds in stems of seven *Achillea* species: *A. cappadocica* (1); *A. setacea* (2); *A. aleppica* (3); *A. coarctata* (4); *A. santolinoides* subsp. *wilhelmsii* (5); *A. santolinoides* (6); *A. arabica* (7).

The PCA3 score plot correlation matrix explained 58.53% of the total variance of the stem data set model (Figure 4). PC1 accounted for 25.54% or the total variance and high correlated with the loadings of dicaffeoylquinic acids, namely 3,4-dicaffeoylquinic (0.655), 3,5-dicaffeoylquinic (0.840), and 4,5-dicaffeoylquinic (0.743) acids. PC2 covered 20.13% of the total variance and correlated with neochlorogenic acid (0.658), chlorogenic acid (0.799), and luteolin-7-glucoside (0.793) and negatively with quercetin (−0.671). PC3 explained 12.83% of the variance and correlated with santin (0.820) and negatively with rutin (−0.541) and luteolin (−0.500). The stems of Achillea spp. were clustered into four groups on the score plot. The first group on the left, negative PC2 and PC3, coupled A. cappadocica (no 1) and A. santolionides (no 6) populations with the lowest amounts of chlorogenic acid, luteolin-7-glucoside. The second group showed a similarity between the stems of A. aleppica (no 3) and A. coarctata (no 4). The third group, consisting of A. setacea (no 2) and A. arabica (no 7) populations, showed a high similarity in the amounts of caffeoylquinic acids in their stems. The arrangement of A. santolinoides subsp. wilhelmsii (no 5) populations coincided with impact of chlorogenic acid and luteolin-7-glucoside. 

Consequently, the multivariate phytochemical patterns complemented the intra- and interspecific diversity of seven *Achillea* spp. according to the contribution of individual caffeoylquinic acids and flavonoids for their clustering in PCA models. Among the species studied, *A. arabica* and *A. setacea* displayed the highest intraspecific chemical diversity. Distant position of *A.* *arabica* was determined by the highest accumulation of mono-caffeoylquinic acids, quercitrin and luteolin-7-glucoside in all plant parts compared to the other species. The arrangement of *A. setacea* populations was confirmed by the high contribution of chlorogenic acid and 3,4- and 3,5-dicaffeoylquinic acids. *Achillea cappadocica*, *A. coarctata*, and *A. santolinoides* showed significant similarity in dicaffeoylquinic acid accumulation in leaves and inflorescences. *Achillea santolinoides* subsp. *wilhelmsii* and *A. alepica* were distinguished from the other species by the lowest levels of caffeoylquinic acids and flavonoids, respectively.

## 3. Discussion

*Achillea* species, compared to many other plant species, are particularly rich in a broad variety of specialized metabolites. The diversity of *Achillea* spp. was assessed on the specific profiles of triterpenes, sterols, polyacetylenes, flavonoids, and phenolic acids, which depend on species-specific characteristics, genotype, stage of ontogenesis and vegetation, as well as climatic and edaphic factors [31,32]. Most chemical studies to date have been performed on *A. millefolium*. However, there are several reports on the composition of phenolic compounds of the species analysed in this study including *A. arabica* [14,30,33,34], *A. santolinoides* subsp. *wilhelmsii*, *A. setacea* [30], and *A. coarctata* [28,33]. Gevrenova et al. [20] tentatively identified various metabolites in the areal parts and roots of *A. aleppica* subsp. *zederbaueri* (Hayek) Hub.-Mor. and *A santolinoides* subsp. *wilhelmsii* areal parts and roots, including 14 hydroxybenzoic and hydroxycinnamic acids, along with 24 glycosyl flavonoids and 12 flavonoid aglycons. 

Among the specialized metabolites identified in *Achillea* spp., phenolic compounds, including caffeoylquinic acids and flavonoids, were considered as a particularly important group because they contribute to the major multifunctional biological activity that may be linked to their antioxidant potential [35]. Caffeoylquinic acids, esters of caffeic acid with quinic acid, have many benefits for therapeutic applications, as were reported about their antioxidant, antibacterial, antiviral, neuroprotective, anticancer, anti-Alzheimer, and anti-diabetes properties [36]. Caffeoylquinic acids, especially 4,5-dicaffeoylquinic acid, have great applications in cosmetic skin whitening products due to their potential for tyrosinase inhibition and tyrosinase transcription gene downregulation [34,37]. It was considered that the biological activities of dicaffeoylquinic acids are attributed to the relative positions of caffeoyl moieties and affected by the structural characteristics of the cyclohexane skeleton [32]. The 3,4- and 4,5-dicaffeoylquinic acids with two adjacent caffeoyl moieties potentially possessed a higher antioxidant capacity than three non-adjacent dicaffeoylquinic acids (1,3-, 1,5- and 3,5-dicaffeoylquinic acids). On the other hand, dicaffeoylquinic acids with non-adjacent caffeoyl moieties exhibited a higher cytoprotective effect than adjacent dicaffeoylquinic acids [38]. Caffeoylquinic acids are photosensitive and chemically unstable, although monocaffeoylquinic acids are far more stable than dicaffeoylquinic acids. Similar to our identification, previous reports indicated that chlorogenic acid was most frequently quantified as the predominant caffeoylquinic acid in *A. arabica*, *A. setacea*, *A. santolinoides* subsp. *wilhelmsii* [14,20,30], *A. coarctata* [28,30], and *A. aleppica* subsp. *zederbaueri* [20] profiles. In addition, 4,5-dicaffeoylquinic acid, 3,5-dicaffeoylquinic acid, and 1,3-dicaffeoylquinic acid were reported to be predominant in *A. arabica*, *A. aleppica* subsp. *zederbaueri*, and *A. santolinoides* subsp. *wilhelmsii,* respectively [20,34]. In our study, 3,4-dicaffeoylquinic, 3,5-dicaffeoylquinic and 4,5-dicaffeoylquinic acids predominated in *A. setacea* and *A. coarctata* extracts, and apparently resulted in the highest antioxidant activity of these species. No 1,3-dicaffeoylquinic acid was detected in the species tested. 

Overall, *Achillea* spp. accumulate higher amounts of caffeoylquinic acids compared to flavonoids [14,28,33,37]. Our results confirmed this trend, as a higher proportion of total caffeoylquinic acids was detected in the inflorescences, leaves, and stems of all studied species except for *A. arabica* inflorescences. Differently than other species, *A. arabica* accumulated higher amounts of flavonoids than caffeoylquinic acids. *A. arabica* exposed an exceptional quantitative profile of flavonoids with the greatest levels of quercetin and luteolin derivatives and flavone santin. Similar to our identification, previous studies reported the higher levels of phenolic compounds in *A. arabica* compared to other *Achillea* spp. [29,34,39,40,41]. Furthermore, *A. arabica* extracts revealed considerable antioxidant activity, suggesting a great contribution of flavonoids to radical scavenging capacity. The radical scavenging potential of flavonoids is determined by the structure of the molecule in which the position and degree of hydroxylation are essential for their antioxidant activity. In this respect, quercetin and its glycosides combine all the structural characteristics of high radical scavenging potential [42]. In addition, the antiradical properties of the extracts might be due to the synergistic action between constituents. A greater synergistic effect was demonstrated for a combination of chlorogenic acid and isoquercitrin in *Sorbus domestica* leaf extracts [43]. This type of interaction possible influenced the antioxidant activity of *A. arabica* extracts. The notable antiradical capacity was also confirmed in *A. cappadocica* extracts, which was associated with high levels of flavonoids and chlorogenic acid. To the best of our knowledge, the profile of *A. cappadocica,* *A. aleppica* and *A. santolinoides* phenolic compounds and the assessment of antioxidant activity were presented for the first time.

Our results are consistent with previous studies that confirmed the presence of rutin, quercetin, luteolin and apigenin in *A. arabica*, *A. coarctata, A*. *setacea*, and *A. santolinoides* subsp. *wilhelmsii* [28,30] raw materials. However, these studies did not present the quantification of flavonoid glycosides in corresponding species, except for isoquercitrin and rutin in *A. coarctata* [28]. In agreement with our results, rutin was identified as the predominant flavonoid in *A. coarctata*. Meanwhile, in another study of *A. coarctata*, rutin was not found at all, although in all cases plant material was collected from Turkey [33]. As far as we know, in our study, the methylated flavone santin was for the first time quantified in the leaves, inflorescences and stems of *A. cappadocica*, *A. setacea*, *A. santolinoides* subsp. *wilhelmsii*, and *A. arabica*. Gevrenova et al. [20] reported the identification of santin in *A. santolinoides* subsp. *wilhelmsii* and *A. aleppica* subsp. *zederbaueri* areal parts from Bulgaria. However, we did not detect santin in *A. aleppica* originating from Turkey. Small amounts of santin was reported in *A. biebersteinii* (*A. arabica*) from Egypt [44]. Santin belongs to lipophilic surface flavonoids with notable antifungal [45] and anti-inflammatory effects [46].

In most previous reports the authors did not specify plant organs, so these data cannot be directly compared with the results presented. Moreover, differences in the comparisons of phenolic compounds composition may be due to inaccuracies in the taxonomic identification of species, harvesting time, methods of extraction, and chemical analysis. According to Zidorn [47] correct species identification is a crucial condition for meaningful phytochemical studies, as well as in comparing data performed by different researchers allegedly working on the same species. Consequently, the comparison of the chemical data has many inconsistencies, but it helps to form an overall picture of the chemical potential of the relevant species’ raw materials.

The chemical diversity of specialized metabolites in vascular plants is a common occurrence affected by different exogenous and endogenous factors that lead to creating new metabolome patterns and formation of intraspecific differences [48]. In this regard, environmental factors, including latitudinal and longitudinal gradients, have been considered to be important in explaining the intraspecific differences of specialized metabolites that may reflect adaptations of plant to local conditions [49]. However, the study of the phenolic profiles of the species over a regional or gradient range will complement the knowledge on the distribution of specialized metabolites in *Achillea* spp. and enrich the information on their biochemical resources. The intraspecific diversity of specialized metabolites is only partly related to the environment. The response of organisms to changes in the abiotic or biotic environment is often genetically controlled [50]. Recently, a new term, chemophenetics, has been proposed to describe an array of specialized metabolites in a given taxon [51]. Chemophenetic information, together with other recognized approaches, contributes to the phenetic description of taxa. The variable phenolic profile patterns across species provide information on the array of distinctive compounds and are an important tool in chemophenetic studies of heterogeneous *Achillea* spp. An example would be the flavonoid santin, which, according to Williams et al. [46], is more frequently occuring in the genus *Achillea* than in other genera of Asteraceae. Santin was reported in 35% species of section *Filipendulinae* and in some taxa of section *Nobilis*, providing its chemotaxonomic significance at the section level. In this study, santin was quantified in species of section *Achillea* (*A. arabica*, *A. cappadocica*, and *A. setacea*) and section *Santolinoideae* (*A. santolinoides* subsp. *wilhelmsii*) [52]. Thus, the new data will complement the information on the prevalence of santin in other sections of the genus *Achillea*. In this context, the use of chemotaxonomic knowledge is appropriate for the selection of wild plant material of relevant taxa that is known to produce desired compounds associated with a particular biological activity or therapeutic effect.

## 4. Materials and Methods

### 4.1. Plant Material Collection and Identification

**Figure 5:**
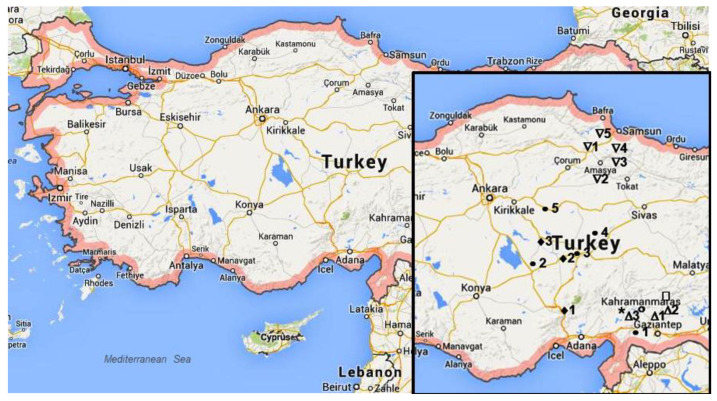
The collection sites of Achillea arabica (Δ), Achillea aleppica (ο), Achillea coarctata (☐), Achillea santalinoides (∗), Achillea cappadocica (♦), Achillea santolinoides subsp. wilhelmsii (●), and Achillea setacea (∇). The number of populations corresponded to No. listed in Table 4.

Plant material of seven species, *Achillea arabica, A. aleppica, A. cappadocica, A. coarctata, A. santalinoides, A. santolinoides* subsp. *wilhelmsii*, and *A. setacea*, representing 30 randomly selected single shoots per population, was collected at full flowering phase during a short period, from 24 to 30 of June 2018. Plants were harvested between 11 h to 13 h to avoid differences in daily variation of metabolism. The plants were collected from 19 wild populations in the provinces of Gaziantep and Kahramanmaraş in the region of Southeastern Anatolia, the provinces of Nevşehir and Niğde in Central Anatolia, and the provinces of Samsun, Amasya and Çorum in the Black Sea region (Figure 5). Sampling sites were geocoded using a Garmin etrex-10 Global Positioning System receiver and exported to map processing software. 

The plant material was dissected into inflorescences, leaves and stems and separately dried at room temperature. The air-dried plant material was mechanically ground to obtain a homogeneous drug powder and stored at 4 °C until extraction. 

Botanical identification of species was performed on morphological characters according to Güner et al. [3], and valid plant names were verified against The Plant List [3,15]. The voucher specimens were deposited in the herbarium of Ondokuz Mayis University, Vocational High School of Bafra (Table 4).

### 4.2. Chemicals

The following solvents were used in the present study: 99.9% acetonitrile, 99.9% methanol, obtained from Sigma-Aldrich (Steinheim, Germany) and 99.8% trifluoroacetic acid supplied from Merck (Darmstadt, Germany). The water was purified using a Millipore Milli-Q apparatus. Analytical and HPLC grade substances: potassium persulphate, 2,2’-azino-bis(3-ethylbenzothiazoline-6-sulfonic acid) diammonium salt (ABTS), caffeic, neochlorogenic, chlorogenic, 4-caffeoylquinic, 3,4-dicaffeoylquinic, 3,5-dicaffeoylquinic, 1,5-dicaffeoylquinic, and 4,5-dicaffeoylquinic acids, rutin, quercitrin, quercetin, isoquercitrin, luteolin-7-glucoside, luteolin-7-rutinoside, luteolin-3,7-diglucoside, apigenin-7-glucoside, and santin were obtained from Sigma-Aldrich; luteolin from Roth GmbH (Karlsruhe, Germany); trolox and apigenin from Fluka Chemika (Buchs, Switzerland). 

### 4.3. Sample Preparation

A precise weight (0.1 g) of each sample plant powder was extracted in 10 mL of 70% methanol in an ultrasonic bath (WiseClean). The extracts were filtered through 0.22 µm syringe filters (Carl RothGmbH & Co. KG, Karlsruhe, Germany) and stored at 4 °C until analysis. 

### 4.4. Antioxidant Activity Assay

The radical scavenging ABTS assay was performed as described by Re et al. [53] with modifications as described by Raudone et al. [54]. 

### 4.5. HPLC Analysis

The chemical composition of phenolic composition was performed by using Waters e2695 Alliance HPLC system coupled with a 2996 PDA detector (Waters, Milford, MA, USA) and the method described by Vilkickyte et al. [55]. Phenolic compounds were separated on an ACE Super C18 (250 mm × 4.6 mm, 3 μm) column (ACT, Aberdeen, UK), operated at a constant temperature of 35 °C. The gradient elution mode consisting of 0.1% (*v*/*v*) trifluoroacetic acid in pure water (A) and acetonitrile (B) was as follows: 0 min, 10% B; 0–40 min, 30% B; 40–60 min, 70% B; 60–64 min, 90% B; 64–70 min, 10%. The flow rate was 0.5 mL/min, and the injection volume was 10 μL. The analytical HPLC-PDA method was validated according to the ICH Q2 (R1) guidelines [56]. Identification was performed by scanning in a range of 200–400 nm wavelengths by comparing spectral data and retention times to those of standard compounds. Representative chromatograms of *Achillea* spp. raw materials are given in Supplementary Figure S1. For quantification, 5–7 points linear calibration curves (r > 0.999) were constructed by plotting the response of each analyte, considered by target concentrations (in the range of 1.6–200.0 μg/mL). Obtained limits of detection (LOD) and quantification (LOQ), determined using the signal-to-noise ratio method, are presented in Supplementary Table S1. Precision values (repeatability of replicates on the same day and intermediate precision on three consecutive days), expressed as percentage relative standard deviations (% RSD) of peak areas did not exceed the 2% threshold. Percent recoveries of studied phenolic compounds were in the acceptable range of 90–110% for our studied concentration levels, showing the trueness of the method.

### 4.6. Statistical Analysis

All investigations were performed in triplicate and expressed as mean ± standard error. An ANOVA, followed by post hoc Duncan ‘s Multiple Range test, was completed to identify significant differences at *p* < 0.05. Pearson’s correlation was analyzed, and *p*-value obtained by checking hypothesis on nonlinear regression was used. Principal component analysis (PCA) was performed considering factors with eigenvalues higher than one. Kaiser–Meyer–Olkin measure of sampling adequacy and Bartlett’s Test were used to test the suitability of the model. The data analysis was performed using SPSS 20 software package.

## 5. Conclusions

The study for the first time reported the fingerprinting of phenolic compounds of areal plant parts of seven *Achillea* spp. together with pronounced antiradical activity of their extracts. Profiles of *Achillea* spp. and plant parts differed in the prevalence of individual caffeoylquinic acids and flavonoids. Among the species studied, *A. arabica* and *A. setacea* populations were displayed by the highest intraspecific chemical diversity, suggesting a greater selection of chemical phenotypes. *Achillea setacea* excelled with the highest total caffeoylquinic acid content, and *A. arabica* accumulated the highest amounts of flavonoids among the species studied. *Achillea santolinoides* subsp. *wilhelmsii* profiles coincided with the lowest content of caffeoylquinic acids, while *A. alepica* with flavonoids. Furthermore, phenolic profile patterns of seven *Achillea* spp. provide information on species-specific individual phenolic compounds that are considered as an important tool in chemophenetic studies of heterogeneous *Achillea* spp. and may be relevant for the selection of target raw materials. The functional benefit of plants extracts was confirmed by their antioxidant activity, which was most pronounced in *A. arabica*, *A. setacea*, and *A. cappadocica*, and which can be considered as potential sources of antioxidants for further applications.

The findings suggest that the high diversity of chemical profiles of *Achillea* spp. leads to a high potential to find new sources of multifunctional phenolic compounds. To further assess and compile the potential of the underutilized *Achillea* spp., it is important to take a broad interdisciplinary approach covering not only phytochemistry, but also ethnopharmacological knowledge, chemotaxonomy, botany, and relevant pharmacological and biological research.

## Figures and Tables

**Table 1:** Mean quantities (µg/g, DM) of phenolic compounds in leaves of *Achillea* spp. and their multivariate comparison among seven wild species.

Compounds	*A. cappadocica*	*A. setacea*	*A. aleppica*	*A. coarctata*	*A.santolinoides* subsp. *wilhelmsii*	*A. santolinoides*	*A. arabica*	*p*-Value
Neochlorogenic acid	^1^608.73 ± 156.58	728.7 ± 111.15	1683.2 ± 84.16	378 ± 18.9	935.4 ± 216.89	519.4 ± 14.99	1824.28 ± 1013.49	0.21
Chlorogenic acid	3347.2 ± 944.48 ^ab1^	8062.76 ± 1279.67 ^abc^	10,020.3 ± 501.02 ^bc^	1973.4 ± 98.67 ^a^	4871.02 ± 1666.55 ^ab^	4038.2 ± 116.57 ^ab^	12,884.98 ± 4288.21 ^c^	<0.05
4-caffeoylquinic acid	193.47 ± 46.78 ^ab^	996.4 ± 368.42 ^b^	873.1 ± 43.66 ^ab^	139.6 ± 6.98 ^a^	508.56 ± 134.31 ^ab^	231.8 ± 6.69 ^ab^	619.5 ± 282.72 ^ab^	<0.05
3,4-dicaffeoylquinic acid	2020.63 ± 627.73 ^ab^	3399.46 ± 1211 ^b^	683.2 ± 34.16 ^a^	1722.2 ± 86.11 ^ab^	570.72 ± 177.14 ^a^	2328.8 ± 67.23 ^ab^	787.68 ± 583.73 ^a^	<0.05
3,5-dicaffeoylquinic acid	7564.07 ± 2067.07 ^ab^	11,570.64 ± 2836.29 ^b^	2758.8 ± 137.94 ^a^	6615.3 ± 330.77 ^ab^	2451.38 ± 853.18 ^a^	5012.2 ± 144.69 ^ab^	4662.28 ± 3081.53 ^ab^	<0.05
1,5-dicaffeoylquinic acid	396.03 ± 79.37 ^b^	21.54 ± 21.54 ^a^	0 ± 0 ^a^	750.4 ± 37.52 ^c^	25.98 ± 16.93 ^a^	404.7 ± 11.68 ^b^	114.28 ± 90.28 ^a^	<0.001
4,5-dicaffeoylquinic acid	1232.77 ± 469.86 ^a^	1062.88 ± 454.86 ^a^	378.8 ± 18.94 ^a^	6615.3 ± 330.77 ^b^	299.82 ± 90.24 ^a^	1210.6 ± 34.95 ^a^	245.5 ± 141.07 ^a^	<0.001
Caffeic acid	34.47 ± 6.73 ^b^	33.56 ± 11.64 ^b^	0.7 ± 0.04 ^a^	21.1 ± 1.06 ^ab^	24.86 ± 6.44 ^ab^	83.6 ± 2.41 ^c^	11.58 ± 6.84 ^ab^	<0.001
Quercitrin	0 ± 0 ^a^	0 ± 0 ^a^	0 ± 0 ^a^	0 ± 0 ^a^	0 ± 0 ^a^	0 ± 0 ^a^	8904.9 ± 5126.48 ^b^	<0.05
Rutin	1785.07 ± 476.65 ^abc^	932.56 ± 531.04 ^ab^	0 ± 0 ^a^	3137.4 ± 156.87 ^c^	130.08 ± 109.22 ^ab^	1019.3 ± 29.42 ^ab^	2115.63 ± 1284.52 ^bc^	<0.05
Quercetin	15.8 ± 7.9 ^a^	23.34 ± 5.97 ^ab^	0 ± 0 ^a^	0 ± 0 ^a^	19.86 ± 0.66 ^ab^	59.2 ± 1.71 ^c^	45.85 ± 15.87 ^bc^	<0.05
Isoquercitrin	1541.5 ± 783.89 ^a^	0 ± 0 ^b^	80.5 ± 4.03 ^b^	199.8 ± 9.99 ^bc^	135.72 ± 80.37 ^bc^	112.1 ± 3.24 ^bc^	1732.24 ± 1629.62 ^a^	<0.05
Luteolin	86.43 ± 43.6 ^b^	129.96 ± 4.9 ^bc^	0 ± 0 ^a^	183.3 ± 9.17 ^c^	114 ± 1.81 ^bc^	151.8 ± 4.38 ^bc^	115.68 ± 39.38 ^bc^	<0.001
Luteolin-7-glucoside	63.4 ± 29.33 ^a^	127.98 ± 35.1 ^b^	703.3 ± 35.17 ^b^	33.8 ± 1.69 ^a^	453.58 ± 215.59 ^b^	39.8 ± 1.15 ^a^	3763.95 ± 3487.18 ^c^	<0.05
Luteolin-7-rutinoside	40.07 ± 20.03 ^a^	88.36 ± 37.22 ^a^	0 ± 0 ^b^	37.8 ± 1.89 ^ab^	271.56 ± 137.1 ^c^	0 ± 0 ^b^	83.5 ± 57.68 ^a^	<0.05
Luteolin-3,7-diglucoside	0 ± 0	0 ± 0	0 ± 0	0 ± 0	0 ± 0	0 ± 0	0 ± 0	-
Apigenin	1.23 ± 1.23	0 ± 0	4 ± 0.2	3.3 ± 0.17	2.78 ± 2.47	0 ± 0	1.73 ± 1.73	0.45
Apigenin-7-glucoside	0 ± 0 ^a^	0 ± 0 ^a^	216.5 ± 10.83 ^b^	0 ± 0 ^a^	158.7 ± 59.39 ^b^	0 ± 0 ^a^	0 ± 0 ^a^	<0.001
Santin	234.7 ± 13.66 ^ab^	784.02 ± 287.25 ^b^	0 ± 0 ^a^	0 ± 0 ^a^	459.94 ± 141.44 ^ab^	0 ± 0 ^a^	267.03 ± 107.43 ^ab^	<0.05

^1^ Values (mean ± SE) of the compounds marked by different letters (a, b) within the row were significantly differed at *p* ≤ 0.05 among species according to the Duncan’s Multiple Range test.

**Table 2:** Mean quantities (µg/g, DM) of phenolic compounds in inflorescences of Achillea spp. and their multivariate comparison among seven wild species.

Compounds	*A. cappadocica*	*A. setacea*	*A. aleppica*	*A. coarctata*	*A.santolinoides* subsp. *wilhelmsii*	*A. santolinoides*	*A. arabica*	*p* Value
Neochlorogenic acid	^1^203.93 ± 19.58	289.58 ± 37.88	230.1 ± 6.64	152.1 ± 4.39	207.96 ± 33.98	228 ± 6.58	682.7 ± 422.66	0.41
Chlorogenic acid	1049 ± 176.38 ^ab^	3249.96 ± 1109.51 ^b^	1405.8 ± 40.58 ^ab^	672.4 ± 19.41 ^a^	839.7 ± 270.28 ^a^	942.1 ± 27.2 ^ab^	2438.85 ± 837.56 ^ab^	<0.05
4-caffeoylquinic acid	99.5 ± 27.15 ^a^	621.26 ± 291.17 ^b^	140.3 ± 4.05 ^ab^	69.1 ± 1.99 ^a^	141.6 ± 33.1 ^ab^	253 ± 7.3 ^ab^	573.05 ± 423.09 ^c^	<0.05
3.4-dicaffeoylquinic acid	589.2 ± 52.04 ^ab^	1822.62 ± 483.73 ^b^	456.7 ± 13.18 ^a^	764.4 ± 22.07 ^ab^	299.58 ± 56.53 ^a^	708.4 ± 20.45 ^sb^	839.8 ± 639.54 ^ab^	<0.05
3.5-dicaffeoylquinic acid	2273.47 ± 302.5 ^ab^	5330.06 ± 1314.69 ^b^	2102.7 ± 60.7 ^ab^	3097.6 ± 89.42 ^ab^	1266.26 ± 329.77 ^a^	1361.4 ± 39.3 ^a^	2125.35 ± 1539.41 ^ab^	<0.05
1.5-dicaffeoylquinic acid	152.63 ± 77.05 ^a^	143.48 ± 51.16 ^a^	63.8 ± 1.84 ^a^	984.6 ± 28.42 ^b^	28.32 ± 19.59 ^a^	104.8 ± 3.03 ^a^	105.8 ± 90.06 ^a^	<0.001
4.5-dicaffeoylquinic acid	295.2 ± 12.65 ^ab^	456.88 ± 95.26 ^b^	292.3 ± 8.44 ^ab^	3097.6 ± 89.42 ^c^	207.94 ± 41.49 ^a^	209.7 ± 6.05 ^a^	242.2 ± 66.74 ^ab^	<0.001
Caffeic acid	21.27 ± 9.02 ^b^	0 ± 0 ^a^	0.1 ± 0 ^a^	25.8 ± 0.74 ^b^	6 ± 4.37 ^a^	49.3 ± 1.42 ^c^	3.25 ± 3.25 ^a^	<0.001
Quercitrin	0 ± 0 ^a^	0 ± 0 ^a^	0 ± 0 ^a^	0 ± 0 ^a^	0 ± 0 ^a^	0 ± 0 ^a^	8487.21 ± 3445.28 ^b^	<0.05
Rutin	155.93 ± 49.75 ^ab^	102.1 ± 49.38 ^a^	0 ± 0 ^a^	93.6 ± 2.7 ^a^	10.1 ± 10.1 ^a^	63.1 ± 1.82 ^a^	265.08 ± 85.94 ^b^	<0.05
Quercetin	60.2 ± 16.18 ^a^	35.58 ± 5.07 ^a^	40 ± 1.15 ^a^	40.2 ± 1.16 ^a^	42.46 ± 8.34 ^a^	54.6 ± 1.58 ^a^	203.7 ± 134.08 ^a^	<0.05
Isoquercitrin	192.63 ± 90.01 ^ab^	513.58 ± 174.94 ^b^	149.3 ± 4.31 ^ab^	24.9 ± 0.72 ^a^	54.14 ± 22.35 ^a^	51.6 ± 1.49 ^a^	182.09 ± 171.89 ^ab^	<0.05
Luteolin	284.27 ± 167.68 ^ab^	723.6 ± 176.02 ^b^	0 ± 0 ^c^	694.7 ± 20.05 ^b^	245 ± 34.84 ^ab^	725.7 ± 20.95 ^b^	560.05 ± 360.27 ^ab^	<0.05
Luteolin-7-glucoside	732.4 ± 253.58 ^a^	1171.72 ± 238.2 ^a^	75.8 ± 2.19 ^a^	181.2 ± 5.23 ^a^	520.06 ± 154.57 ^a^	978.5 ± 28.25 ^a^	5646.13 ± 3185.87 ^b^	<0.05
Luteolin-7-rutinoside	95.77 ± 33.1 ^a^	156.86 ± 68.58 ^a^	37.8 ± 1.09 ^a^	0 ± 0 ^a^	170.38 ± 46.78 ^a^	76.9 ± 2.22 ^a^	217.98 ± 169.76 ^a^	<0.05
Luteolin-3.7-diglucoside	0 ± 0 ^a^	531.26 ± 260.77 ^b^	0 ± 0 ^a^	0 ± 0 ^a^	0 ± 0 ^a^	0 ± 0 ^a^	596.8 ± 596.8 ^b^	<0.05
Apigenin	43.6 ± 26.55 ^a^	47.72 ± 30.84 ^a^	0 ± 0 ^b^	2.3 ± 0.07 ^b^	0 ± 0 ^b^	29.2 ± 0.84 ^ab^	6.88 ± 4.88 ^b^	<0.05
Apigenin-7-glucoside	138 ± 97.09 ^a^	137.66 ± 80.47 ^a^	18.7 ± 0.54 ^b^	0 ± 0 ^b^	41.3 ± 12.09 ^b^	99.3 ± 2.87 ^ab^	13.28 ± 13.28 ^b^	<0.05
Santin	229.07 ± 10.36 ^a^	548.8 ± 190.9 ^b^	0 ± 0 ^c^	0 ± 0 ^c^	228.32 ± 16.21 ^a^	0 ± 0 ^c^	321.7 ± 90.89 ^ab^	<0.05

^1^ Values (mean ± SE) of the compounds marked by different letters (a, b, c) within the row were significantly differed at *p* ≤ 0.05 among species according to the Duncan’s Multiple Range test.

**Table 3:** Mean quantities (µg/g, DM) of phenolic compounds in stems of *Achillea* spp. and their multivariate comparison among seven wild species.

Compounds	*A. cappadocica*	*A. setacea*	*A. aleppica*	*A. coarctata*	*A.santolinoides* subsp. *wilhelmsii*	*A. santolinoides*	*A. arabica*	*p* Value
Neochlorogenic acid	^1^198.13 ± 15.83 ^a^	277.6 ± 56.41 ^ab^	430.5 ± 12.43 ^b^	169.6 ± 4.9 ^a^	264.88 ± 56.6 ^ab^	223 ± 6.44 ^ab^	347.18 ± 122.04 ^ab^	<0.05
Chlorogenic acid	670.97 ± 121.5 ^a^	1554.64 ± 340.63 ^b^	1901.4 ± 54.89 ^b^	554.9 ± 16.02 ^a^	2044.22 ± 1086.49 ^b^	1001.4 ± 28.91 ^b^	1992.4 ± 709.1 ^b^	<0.05
4-caffeoylquinic acid	55.03 ± 4.08 ^a^	354.58 ± 149.37 ^b^	280.6 ± 8.1 ^b^	51.1 ± 1.48 ^a^	252.74 ± 86.63 ^b^	87.7 ± 2.53 ^a^	269.23 ± 197.21 ^b^	<0.05
3.4-dicaffeoylquinic acid	388.8 ± 94.75 ^a^	664.18 ± 206.38 ^b^	361.8 ± 10.44 ^a^	441.8 ± 12.75 ^ab^	219.84 ± 63.56 ^a^	409.4 ± 11.82 ^ab^	318.25 ± 226.39 ^a^	<0.05
3.5-dicaffeoylquinic acid	1557.77 ± 152.48	1583.34 ± 360.82	1167.4 ± 33.7	1916.9 ± 55.34	1175.4 ± 423.71	1210.6 ± 34.95	1165.95 ± 745.73	0.84
1.5-dicaffeoylquinic acid	169.8 ± 17.51 ^b^	85.2 ± 10.66 ^a^	40.2 ± 1.16 ^a^	524 ± 15.13 ^c^	102.58 ± 30.18 ^ab^	105.6 ± 3.05 ^ab^	54 ± 36.29 ^a^	<0.001
4.5-dicaffeoylquinic acid	273.07 ± 30.27 ^bc^	177.18 ± 42.64 ^abc^	433.6 ± 12.52 ^c^	296.4 ± 8.56 ^bc^	122.96 ± 28.15 ^a^	211.5 ± 6.11 ^abc^	131.95 ± 79.11 ^ab^	<0.001
Caffeic acid	2.47 ± 1.18 ^a^	8.12 ± 1.01 ^a^	25.8 ± 0.74 ^b^	2.6 ± 0.08 ^a^	8.24 ± 2.26 ^a^	31.5 ± 0.91 ^b^	10.85 ± 9.06 ^a^	<0.001
Quercitrin	0 ± 0 ^a^	0 ± 0 ^a^	0 ± 0 ^a^	0 ± 0 ^a^	0 ± 0 ^a^	0 ± 0 ^a^	2442.38 ± 1407.88 ^b^	<0.05
Rutin	204.03 ± 16.38 ^a^	366.86 ± 219.79 ^a^	0 ± 0 ^b^	1732.2 ± 50 ^c^	46.1 ± 28.38 ^b^	63.5 ± 1.83 ^b^	355.8 ± 213.21 ^a^	<0.001
Quercetin	54.57 ± 5.93 ^b^	29.84 ± 2.37 ^a^	24.1 ± 0.7 ^a^	24.8 ± 0.72 ^a^	21 ± 0.7 ^a^	76.6 ± 2.21 ^c^	47.7 ± 8.31 ^b^	<0.001
Isoquercitrin	476.67 ± 111.16 ^bc^	0 ± 0 ^a^	0 ± 0 ^a^	0 ± 0 ^a^	35.2 ± 23.64 ^a^	720.5 ± 20.8 ^c^	303.23 ± 303.23 ^ab^	<0.001
Luteolin	74.03 ± 37.07	45.4 ± 27.81	129.6 ± 3.74	111.7 ± 3.22	48.58 ± 30	118.5 ± 3.42	100.93 ± 34.4	0.21
Luteolin-7-glucoside	6.9 ± 3.86 ^a^	18.96 ± 4.86 ^a^	86.4 ± 2.49 ^ab^	13.5 ± 0.39 ^a^	197.36 ± 74.13 ^b^	5.1 ± 0.15 ^a^	198.55 ± 114.2 ^b^	<0.05
Luteolin-7-rutinoside	203.87 ± 93.88 ^a^	135 ± 14.8 ^ab^	105.1 ± 3.03 ^ab^	32.9 ± 0.95 ^b^	46.58 ± 18.63 ^b^	179.9 ± 5.19 ^ab^	162.93 ± 90.55 ^ab^	0.02
Luteolin-3.7-diglucoside	0 ± 0	0 ± 0	0 ± 0	0 ± 0	0 ± 0	0 ± 0	0 ± 0	-
Apigenin	0 ± 0	0 ± 0	0 ± 0	0 ± 0	0 ± 0	0 ± 0	0 ± 0	-
Apigenin-7-glucoside	0 ± 0	0 ± 0	0 ± 0	0 ± 0	0 ± 0	0 ± 0	0 ± 0	-
Santin	214.9 ± 6.37 ^a^	247.48 ± 15.54 ^a^	0 ± 0 ^b^	0 ± 0 ^b^	243.72 ± 22.41 ^a^	0 ± 0 ^b^	223.08 ± 5.52 ^a^	<0.001

^1^ Values (mean ± SE) of the compounds marked by different letters (a, b, c) within the row were significantly differed at *p* ≤ 0.05 among species according to the Duncan’s Multiple Range test.

**Table 4:** Voucher numbers, geographical data of collection sites and habitats of the studied *Achillea* species.

Species	Population	Voucher Number	Province, Geographical Region	Latitude (°N)	Longitude (°E)	Elevation (m a.s.l.)	Habitat
*A. cappadocica*	1	BMYO #AC1	Niğde, Southern Anatolia	37. 54	34.54	2057	Conifer woodland
	2	BMYO #AC2	Nevşehir, Central Anatolia	38.28	34.40	1596	Highland meadow
	3	BMYO #AC3	Nevşehir, Central Anatolia	38.39	34.29	937	Highland meadow
*A. setacea*	1	BMYO #AS1	Çorum, Black Sea	40.53	35.13	1012	Barren mountain slope
	2	BMYO #AS2	Amasya, Black Sea	40.47	35.25	914	Barren mountain slope
	3	BMYO #AS3	Çorum, Black Sea	40.56	35.39	1197	Barren mountain slope
	4	BMYO #AS4	Amasya, Black Sea	41.04	42.13	1117	Stony roadside
	5	BMYO #AS5	Samsun, Black Sea	41.09	35.11	664	Stony roadside
*A. aleppica*	1	BMYO #Aa1	Gaziantep, Southeastern Anatolia	37.09	37.24	668	Calcareous mountainside
*A. coarctata*	1	BMYO#Aa2	Gaziantep, Southeastern Anatolia	37.16	37.46	446	Stony roadside
*A.santolinoides* subsp. *wilhelmsii*	1	BMYO #AW1	Gaziantep, Southeastern Anatolia	36.52	36.59	1054	High altitude stony land
	2	BMYO #AW2	Niğde, Central Anatolia	38.21	34.22	1384	Stony calcareous areas
	3	BMYO #AW3	Niğde, Central Anatolia	38.25	34.51	1726	Stony calcareous area
	4	BMYO #AW4	Niğde, Central Anatolia	38.27	34°.59	1661	Calcareous stony areas
	5	BMYO #AW5	Nevşehir, Central Anatolia	39.38	35.54	1139	Calcareous stony area
*A. santolinoides*	1	BMYO #Aa3	Gaziantep, Southeastern Anatolia	37.20	37.03	520	Stony roadside
*A. arabica*	1	BMYO * #AA54	Kahramanmaraş, Southeastern Anatolia	36.58	37.24	975	Conifer woodland
	2	BMYO # AA55	Gaziantep, South-eastern Anatolia	37.06	37.38	682	Conifer woodland
	3	BMYO # AA56	Gaziantep, Southeastern Anatolia	37.01	37.06	1276	Conifer woodland

* Bafra Meslek Yüksek Okulu (Vocational High School of Bafra).

## Data Availability

Data is contained within the article or supplementary material.

## Supplementary Materials

The following supporting information can be downloaded at: https://www.mdpi.com/article/10.3390/plants11030447/s1, Figure S1: Representative HPLC-PDA chromatograms (λ = 330 nm) of *Achillea* spp. *(A. setacea*) (A) leaves, (B) stems, and (C) inflorescences, showing separation of: 1—neochlorogenic acid, 2—chlorogenic acid, 3—4-caffeoylquinic acid, 4—luteolin-3.7-diglucoside, 5—caffeic acid, 6—luteolin-7-rutinoside, 7—rutin, 8—luteolin-7-glucoside, 9—isoquercitrin, 10—4.5-dicaffeoylquinic acid, 11—apigenin-7-glucoside, 12—1.5-dicaffeoylquinic acid, 13—3.5-dicaffeoylquinic acid, 14—3.4-dicaffeoylquinic acid, 15—luteolin, 16 —quercetin, 17—apigenin, 18—santin.; Table S1: HPLC-PDA method identification and quantification parameters.
