# Supplementary material for: Distribution of Phenolic Compounds and Antioxidant Activity in Plant Parts and Populations of Seven Underutilized Wild Achillea Species
Source: Plants (Basel). 2022 Feb 6;11(3):447. doi: 10.3390/plants11030447 (PMC8839896; doi:10.3390/plants11030447)
Supplement: Supplementary file 1 [file plants-11-00447-s001.zip › plants-1575263-supplementary.pdf]

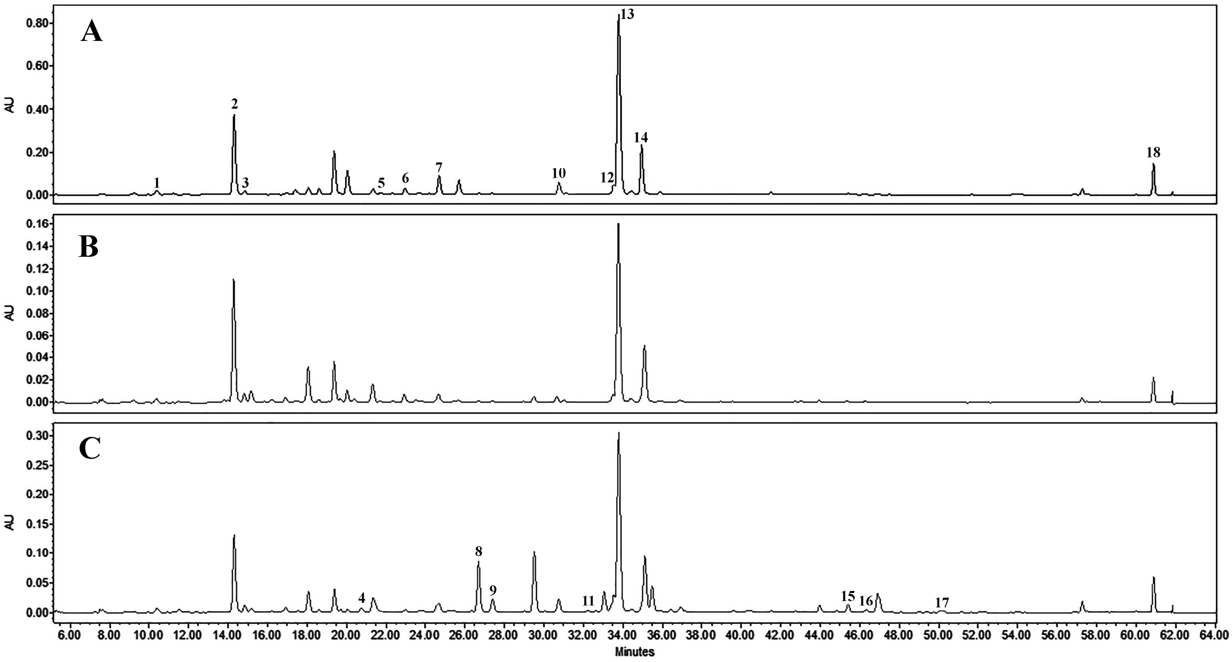

Figure S1. Representative HPLC-PDA chromatograms (λ = 330 nm) of Achillea spp. (*A. setacea*) (A) leaves, (B) stems, and (C) inflorescences, showing separation of: 1—neochlorogenic acid, 2—chlorogenic acid, 3—4-caffeoylquinic acid, 4—luteolin-3.7-diglucoside, 5—caffeic acid, 6—luteolin-7-rutinoside, 7—rutin, 8—luteolin-7-glucoside, 9—isoquercitrin, 10—4.5-dicaffeoylquinic acid, 11—apigenin-7-glucoside, 12—1.5-dicaffeoylquinic acid, 13—3.5-dicaffeoylquinic acid, 14—3.4-dicaffeoylquinic acid, 15—luteolin, 16 —quercetin, 17—apigenin, 18—santin.

Table S1. HPLC-PDA method identification and quantification parameters.

Compound	Calibration curve	Coefficient of determination (r^2)	LOD (μg/mL)	LOQ (μg/mL)
Neochlorogenic acid	f(x) = 42500x - 15000	0.99970	0.32	0.97
Chlorogenic acid	f(x) = 48500x + 47200	0.99927	0.27	0.83
4-caffeoylquinic acid	f(x) = 56700x - 91900	0.99993	0.11	0.33
3.4-dicaffeoylquinic acid	f(x) = 56700x - 6790	0.99999	0.03	0.09
3.5-dicaffeoylquinic acid	f(x) = 79100x - 36900	0.99990	0.09	0.28
1.5-dicaffeoylquinic acid	f(x) = 70500x - 23900	0.99937	0.65	1.57
4.5-dicaffeoylquinic acid	f(x) = 40400x - 9600	0.99998	0.05	0.15
Caffeic acid	f(x) = 112000x + 24100	0.99974	0.37	1.25
Quercitrin	f(x) = 39500x + 3580	0.99961	0.23	0.71
Rutin	f(x) = 30300x + 3050	0.99993	0.10	0.30
Quercetin	f(x) = 74800x - 70600	0.99984	0.13	0.38
Isoquercitrin	f(x) = 39100x + 2560	0.99995	0.13	0.39
Luteolin	f(x) = 25200x - 26000	0.99931	0.48	1.44
Luteolin-7-glucoside	f(x) = 54300x + 1040	0.99998	0.05	0.15
Luteolin-7-rutinoside	f(x) = 40300x - 3980	0.99999	0.03	0.08
Luteolin-3.7-diglucoside	f(x) = 31200x + 19100	0.99929	0.31	0.94
Apigenin	f(x) = 90100x + 9770	0.99997	0.03	0.10
Apigenin-7-glucoside	f(x) = 68600x - 3820	0.99992	0.06	0.17
Santin	f(x) = 64700x - 132000	0.99975	0.47	1.41
